# A combined signature of glycolysis and immune landscape predicts prognosis and therapeutic response in prostate cancer

**DOI:** 10.3389/fendo.2022.1037099

**Published:** 2022-10-21

**Authors:** Tao Guo, Jian Wang, Shi Yan, Xiangyu Meng, Xiaomin Zhang, Shuang Xu, Shancheng Ren, Yuhua Huang

**Affiliations:** ^1^ Department of Urology, The First Affiliated Hospital of Soochow University, Suzhou, Jiangsu Province, China; ^2^ Department of Urology, Shanghai Changhai Hospital, Shanghai, China; ^3^ Department of Urology , The First Affiliated Hospital of Nanjing Medical University, Nanjing, China; ^4^ Department of Urology, Shanghai Changzheng Hospital, Shanghai, China

**Keywords:** prostate cancer, glycolysis, tumor microenvironment, prognosis, therapeutic sensitivity

## Abstract

Prostate cancer (PCa) is a common malignancy that poses a major threat to the health of men. Prostate-specific antigen (PSA) and its derivatives, as FDA-approved detection assays, are insufficient to serve as optimal markers for patient prognosis and clinical decision-making. It is widely acknowledged that aberrant glycolytic metabolism in PCa is related to tumor progression and acidifies the tumor microenvironment (TME). Considering the non-negligible impacts of glycolysis and immune functions on PCa, we developed a combined classifier in prostate cancer. The Glycolysis Score containing 19 genes and TME Score including three immune cells were created, using the univariate and multivariate Cox proportional hazards model, log-rank test, least absolute shrinkage and selection operator (LASSO) regression analysis and the bootstrap approach. Combining the glycolysis and immunological landscape, the Glycolysis-TME Classifier was then constructed. It was observed that the classifier was more accurate in predicting the prognosis of patients than the current biomarkers. Notably, there were significant differences in metabolic activity, signaling pathways, mutational landscape, immunotherapeutic response, and drug sensitivity among the Glycolysis^high^/TME^low^, Mixed group and Glycolysis^low^/TME^high^ identified by this classifier. Overall, due to the significant prognostic value and potential therapeutic guidance of the Glycolysis-TME Classifier, we anticipate that this classifier will be clinically beneficial in the management of patients with PCa.

## Introduction

Prostate cancer (PCa) is one of the most prevalent malignancies affecting the male genitourinary system worldwide. According to the American Cancer Society, prostate cancer has the highest incident cases among cancers in males, accounting for 27% of diagnoses for tumors (1). Although localized or regional prostate cancer has a 5-year survival rate over 99%, advanced prostate cancer is considered incurable (1, 2). Androgen deprivation therapy is the backbone of therapy for advanced prostate cancer (3), however, resistance occurs over time and PCa eventually progresses into castration-resistant prostate cancer (CRPC) (4). CRPC has a poor prognosis, with a median survival after the onset of castration resistance ranging approximately between 9 and 30 months (5). Currently, the most commonly used drugs for CRPC include docetaxel, enzalutamide and abiraterone acetate albeit the cure remains elusive (6). The prognosis of PCa is greatly influenced by an early diagnosis. With the development of methodologies, diagnostic and prognostic methods based on radiomics, genomics and radiogenomics have improved risk stratification and clinical decision making (7). Advances in the detection of biomarkers could help identify potential false-negative cases (8). However, there are still limitations in terms of prognostic prediction regarding existing strategies. Therefore, developing a more accurate prognostic model with the aid of new technologies, such as next-generation sequencing (NGS), is necessary to further guide patients into precise drug administration and improve their prognostic survival.

As PCa progresses, metabolic reprogramming constantly occurs from prostatic intraepithelial neoplasia to metastatic CRPC (9). The Warburg effect is a prominent tumor characteristic that favorably utilizes aerobic glycolysis over mitochondrial oxidative phosphorylation to generate lactate metabolites and energy, even under oxygen-enriched conditions (10). Although glycolysis is not a metabolic signature of primary PCa (11), advanced PCa has been closely associated with an enhanced glycolytic phenotype. Emerging data have indicated that elevated glycolysis promotes metastasis, invasion and even resistance to chemotherapeutics (12). Therefore, it is increasingly evident that interrupting cancer glycolysis will possibly inhibit tumor development to provide therapeutic opportunities (13). In addition to directly impact tumor cell survival, glycolysis alters the tumor microenvironment (TME), especially the immune cells, including the activation of natural killer (NK) cells, CD4^+^ T cells (14, 15), the generation of memory CD8^+^ T cells (Tm) (15), macrophage polarization (16) and Treg suppression function (17), etc. The accumulation of lactic acid by enhanced glycolytic activity acidifies the TME and further contributes to immunosuppression *in vivo* (18).

Previous studies have established glycolytic gene expression signatures in breast cancer (19), ovarian cancer (20), lung adenocarcinoma (21), clear cell renal cell carcinoma (22), colon adenocarcinoma (23) and osteosarcoma (24). However, no research has focused on the combination signature of glycolysis and TME for clinical categorization and personalized therapy. We systematically integrated both into the Glycolysis-TME signature and innovatively improved the robustness of the classifier under the bootstrap algorithm. Consequently, prognosis and therapeutic response in patients with prostate adenocarcinoma (PRAD) were accurately predicted by the Glycolysis-TME Classifier, which improved our understanding of the impact of TME on tumor ecology under glycolytic reprogramming.

## Materials and methods

### Data acquisition

The Global Data Consortium (GDC) data portal (https://portal.gdc.cancer.gov/), UCSC Xena datasets (http://xena.ucsc.edu/), and cBioPortal (https://www.cbioportal.org/) were used to acquire The Cancer Genome Atlas (TCGA) cohort of mRNA normalized expression data, tumor mutation burden (TMB), DNA methylation data, copy number variations (CNVs) and clinical information. We used cBioPortal to download the MSKCC project (MSKCC, Cancer Cell 2010) transcriptome data and prognostic survival data from the Memorial Sloan Kettering Cancer Centre project (MSKCC, Cancer Cell 2010). In addition, GSE54460, GSE70769 and single-cell data of GSE137829 were obtained from the Gene Expression Omnibus (GEO) (https://www.ncbi.nlm.nih.gov/geo/). The following public databases were used: GSCA (http://bioinfo.life.hust.edu.cn/GSCA/#/), GSCALite (http://bioinfo.life.hust.edu.cn/web/GSCALite/) (25), Metascape (http://metascape.org/) (26) and Proteomaps (https://proteomaps.net/) (27).

### Identification of differentially expressed genes in glycolysis-related genes

A total of 200 GRGs were initially obtained from Hallmark gene sets (“HALLMARK_GLYCOLYSIS”) in the Molecular Signatures Database (MSigDB) (28). Using the “limma” package (29), we explored DEGs with P values<0.05 between prostate cancer and paracancerous samples, and the heatmap displaying prognosis-related DEGs was produced using the “pheatmap” package.

### Gene ontology and kyoto encyclopedia of genes and genomes analysis

Based on R “clusterProfiler” (30), We performed GO and KEGG analyses to investigate the functions and pathways associated with gene sets and the cut-off threshold for filtering required both P-values and Q-values to be less than 0.05. All three ontologies including “biological process” (BP), “cellular component” (CC), “molecular function” (MF) and KEGG pathways were observed only 10 items respectively with the “enrichplot” package.

### Construction of Glycolysis score

Univariate Cox proportional hazards regression analysis was used to screen prognosis-related DEGs (p< 0.05). To further refine the model, we identified 19 of these genes using the least absolute shrinkage and selection operator (LASSO) analysis. Resampling 1000 times of TCGA-PRAD samples using the bootstrap (“boot” package) was to obtain a stable signature: The coefficients of multivariate regression were divided by the standard deviation computed with the bootstrap procedure, and then multiplied by the corresponding gene expressions. Eventually, all the values were summed up:


$$\text{Glycolysis Score}=\sum^{}\frac{\text{Coef i}*\text{Exp i}}{\text{Bootstrap(SD)}}$$

(Coef i: coefficients of multivariate regression; Exp i: expressions of each gene; Bootstrap(SD): standard deviations computed with bootstrap)

### Construction of TME Score

For each sample, immune cell landscapes were deduced using the CIBERSORT method, and immune cells associated with prognosis were identified using the Kaplan-Meier (KM) survival analysis. Ultimately, a similar method to the Glycolysis Score but converted to the opposite number was applied to construct the TME Score:


$$\text{TME Score}=\sum^{}\frac{-\text{Coef i}*\text{C j}}{\text{Bootstrap(SD)}}$$

(Coef i: coefficients of multivariate regression; C j: the abundance of prognostically relevant immune cells; Bootstrap(SD): standard deviations computed with bootstrap)

### Construction and validation of Glycolysis-TME Classifier

We divided the cohort into two groups with high and low scores (Glycolysis^high^ and Glycolysis^low^) based on the median of Glycolysis Score. In a similar way, the cohort was divided into TME^high^ and TME^low^ by the median of TME Score. Then, the two types of groupings obtained by Glycolysis Score and TME Score were integrated to construct the Glycolysis-TME Classifier. Eventually, there were four groups in the TCGA-PRAD cohort: the Glycolysis^high^/TME^low^, Glycolysis^high^/TME^high^, Glycolysis^low^/TME^high^, and Glycolysis^low^/TME^low^. To create fewer groups, Glycolysis^high^/TME^high^ and Glycolysis^low^/TME^low^ were further combined into the Mixed group. We also validated the prognostic power of this classifier in the MSKCC cohort, GSE54460 and GSE70769, and principal component analysis (PCA) was performed to compare distinct transcriptional profiles among categorized subgroups.

### Gene set enrichment analysis and fast gene set enrichment analysis

To identify differences in molecular functional characteristics among different subgroups, we utilized the R package “clusterProfiler” for GSEA analysis. Furthermore, Fast Gene Set Enrichment Analysis (FGSEA) was also implemented *via* the “fgsea” package for fast functional analysis concerning Gene Ontology.

### Weighted gene co-expression network analysis and investigation of gene modules

Following the WGCNA tutorial (31), a beta power (β = 12) was selected at a soft threshold of 0.85 (R “WGCNA”). The underlying mechanisms among the groups were revealed by the constructed network module. Further, we uploaded the genes in the selected module to Metascape for pathway and process enrichment analysis and protein-protein interaction (PPI) analysis.

### Analysis of copy number variations and DNA methylation

Heterozygosity and homozygosity for amplifications and deletions were incorporated to assess the frequency of CNVs for genes in the Glycolysis Score. We further evaluated the relationship between CNVs and transcriptional expression levels with the Spearman correlation analysis. In the meantime, log-rank tests were performed to evaluate the survival differences among the different groups.

Similarly, we applied the t-test for the differential methylation analysis of tumors and normal adjacent tissues and tested the association between mRNA expression levels and methylation levels using the Spearman correlation analysis. The prognostic relevance of methylation levels for each gene was identified using the Cox Proportional-Hazards model.

### Single-cell analysis

We utilized Seurat (R package Seurat) for dimension reduction and clustering analysis on two samples from GSE137829. Cells with the number of detected genes less than 200 or more than 5000 gene numbers were filtered out. The mitochondrial and hemoglobin gene proportion was limited to less than 20% and 3%, respectively. AddModuleScore of Seurat was then used to obtain the composite expression score of gene sets.

### Analysis of somatic variants

Visualization of genomic profile diagram was to observe the frequency and distribution of mutations among various groups using the “maftools” R package (32). In addition, we evaluated TMB variations amongst three subgroups based on somatic mutation data.

### Prediction of drug sensitivity

GSCA database was used to analyze the correlation between gene expression and drug sensitivity, using Genomics of Drug Sensitivity in Cancer (GDSC) and Cancer Therapeutics Response Portal (CTRP) datasets as the training set. The “oncoPredict” package (33) was subsequently used to evaluate the sensitivity of individual samples to various drugs based on the CTRP dataset. We used the half-maximal inhibitory concentration (IC50) to measure the drug response.

### Statistical analysis

All data analysis and graph visualization were conducted using the R program (version 4.1.3). For the comparison of continuous variables between two groups, differences between normally distributed variables were evaluated by Student’s t-test, and differences between non-normally distributed variables were evaluated by Wilcoxon rank-sum test. Chi-squared test was applied to analyze the difference in categorical variables including clinicopathological features between Glycolysis^high^/TME^low^ and Glycolysis^low^/TME^high^. TMB, the expression of immune checkpoints and estimated IC50 of drugs among the three groups were compared using the Kruskal-Wallis test. The Spearman or Pearson correlation analysis was implemented to assess the relationship between two variables. We performed survival analysis using Cox proportional hazards regression models, and log-rank tests were carried out to compare differences in survival. Two-sided P values less than 0.05 were considered statistically significant.

## Results

### Establishment of Glycolysis score and TME Score

**Figure 1:**
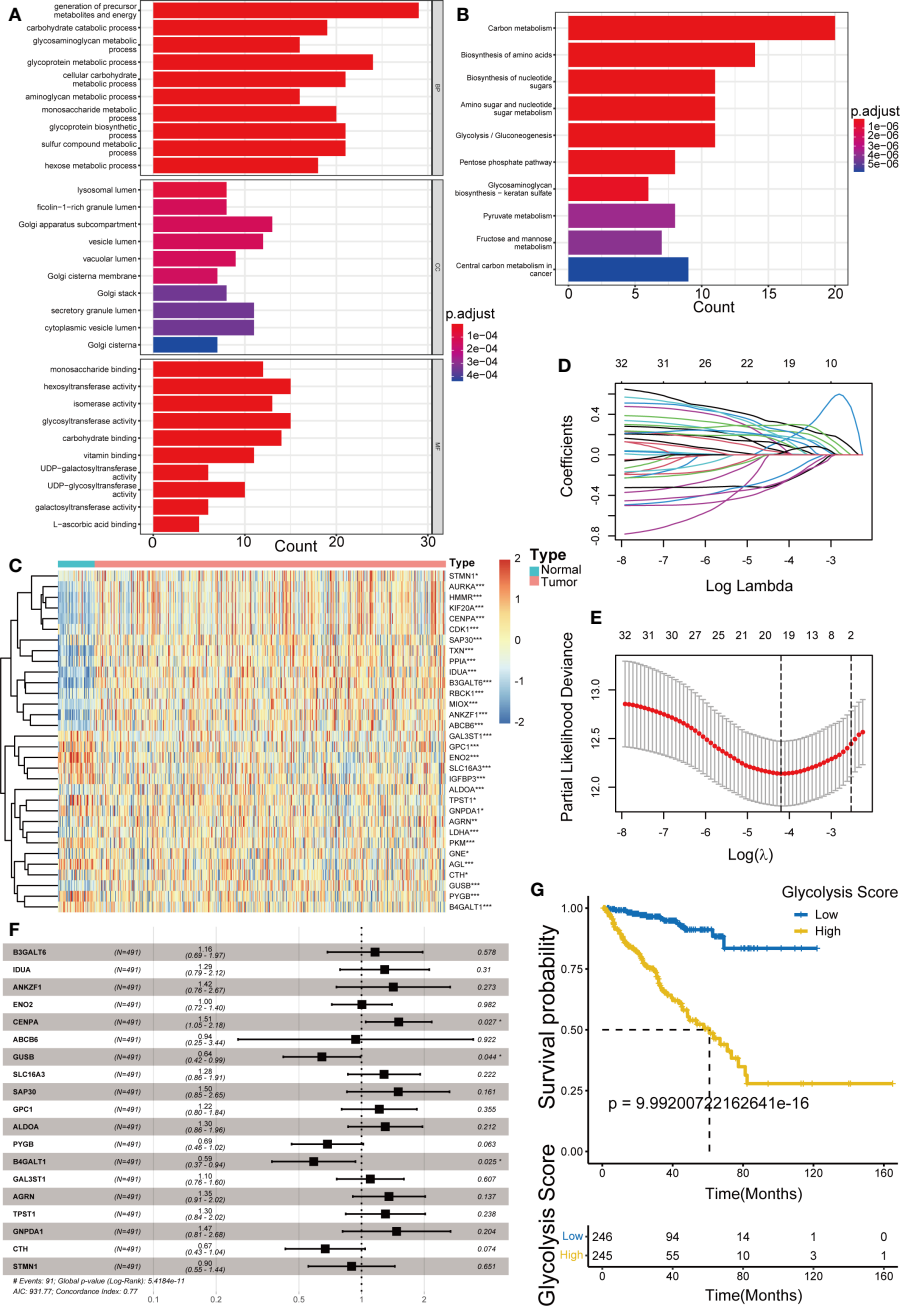
Establishment of Glycolysis Score. **(A, B)** Gene Ontology (GO) and Kyoto Encyclopedia of Genes and Genomes (KEGG) enrichment analysis of the glycolysis-related DEGs. **(C)** Heatmap showing the difference of prognostic glycolysis-related genes (GRGs) between tumor and normal tissues. **(D, E)** The least absolute shrinkage and selection operator (LASSO) Cox regression analysis for the prognosis-related GRGs. **(F)** The multivariable Cox regression analysis of 19 GRGs in the Glycolysis Score. **(G)** Kaplan–Meier analysis for disease-free survival (DFS) of patients from TCGA-PRAD between groups defined by the Glycolysis Score. *p< 0.05; ***p< 0.001.

A simplified flow chart summarizing the pipeline is provided in **Supplementary Figure 1**. The results of difference analysis of the GRGs between prostate cancer and paracancerous tissues, revealed that 131 of these DEGs were upregulated or downregulated in PRAD (p< 0.05). GO and KEGG analyses of these genes revealed a strong association with carbon and other anabolic metabolism (**Figures 1A, B**). Further, 32 genes associated with disease-free survival (DFS), as the outcome of interest, were identified using the univariate Cox regression analysis (p< 0.05) (**Supplementary Figure 2**), and the expression heatmap of these genes between tumor and normal samples was presented in **Figure 1C**. The LASSO Cox regression analysis, bootstrap procedure and multivariable Cox regression analyses were performed to construct a risk signature containing 19 genes (B3GALT6, IDUA, ANKZF1, ENO2, CENPA, ABCB6, GUSB, SLC16A3, SAP30, GPC1, ALDOA, PYGB, B4GALT1, GAL3ST1, AGRN, TPST1, GNPDA1, CTH and STMN1) (**Figures 1D–F**). The K-M survival analysis for DFS in patients from TCGA-PRAD revealed significant differences among groups (p< 0.001) (**Figure 1G**). Patients with high-risk scores had a higher disease recurrence rate than those with low-risk scores (p< 0.05, log-rank test).

**Figure 2:**
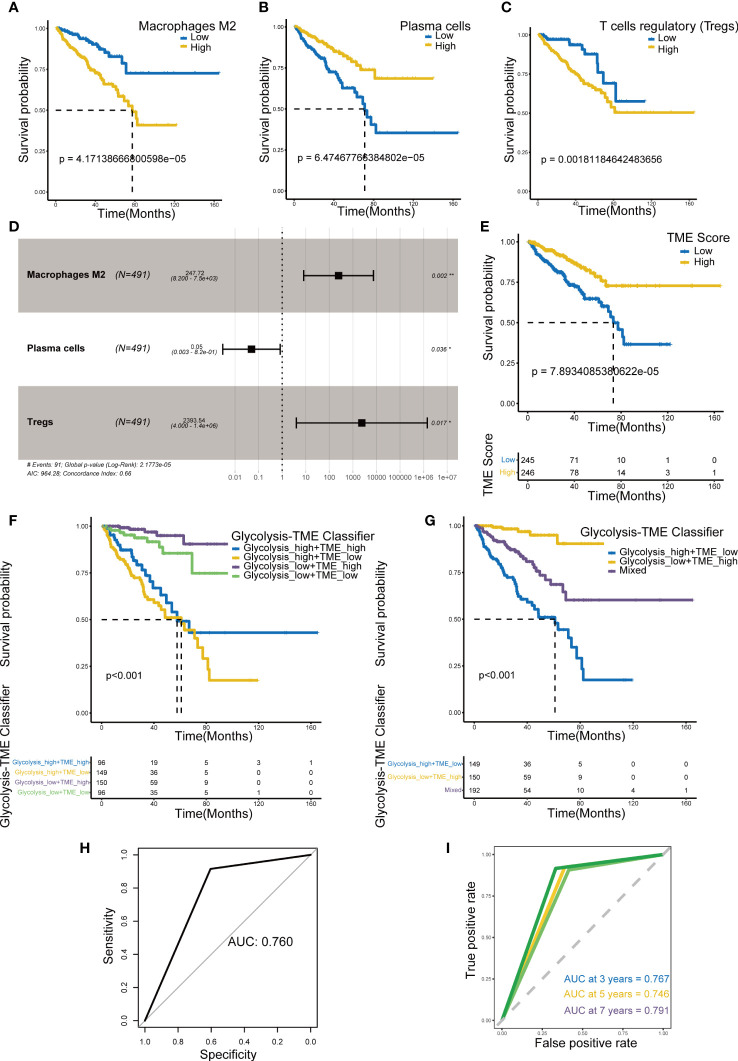
Establishment of TME Score and Glycolysis-TME Classifier. **(A-C)** Prognosis-associated immune cells including M2 macrophages, plasma cells and regulatory T cells. **(D)** The multivariable Cox regression analysis of 3 TME cells in the TME Score. **(E)** Kaplan–Meier analysis for DFS of patients from TCGA-PRAD between groups defined by the TME Score. **(F, G)** Kaplan–Meier analysis for DFS of patients from TCGA-PRAD among groups defined by the Glycolysis-TME Classifier. **(H, I)** ROCs for total, 3-year, 5-year and 7-year survival prediction. *p< 0.05; **p< 0. 01.

The relative scores of immune cells in PCa samples were calculated by the CIBERSORT approach. Subsequently, KM curves revealed prognosis-related infiltrating immune cells including macrophage M2, plasma cells and regulatory T cells (Tregs) (**Figures 2A–C**) (p< 0.005). In addition, we established the TME Score (**Figure 2D**) for these three fractions of cell *via* bootstrap resampling and multivariate Cox regression analysis. This score also demonstrated strong prognostic predictive potential (**Figure 2E**).

### Prognostic value of Glycolysis-TME Classifier

Based on the Glycolysis Score and TME Score established above (**Supplementary Tables 1**, **2**), we combined them to construct the Glycolysis-TME classifier, which divided the cohort into four groups: the Glycolysis^high^/TME^low^, Glycolysis^high^/TME^high^, Glycolysis^low^/TME^high^ and Glycolysis^low^/TME^low^. The statistical difference in prognosis among the groups distinguished by the classifier was nevertheless statistically different (**Figure 2F**), with the Glycolysis^low^/TME^high^ having a better prognosis in DFS and Glycolysis^high^/TME^low^ the worst. We further merged the Glycolysis^high^/TME^high^ and Glycolysis^low^/TME^low^ to reduce the number of groups, which also demonstrated significant results in prognostic prediction (**Figure 2G**). After the merging, it can be observed that the classifier distinguished different groups more clearly (**Supplementary Figures 3A, B**). **Figures 2H, I** demonstrated that the classifier has good predictive performance using receiver-operating characteristic (ROC) analysis, with the area under the curve ranging from 0.746 to 0.791 for 3, 5, 7 years and total, all of which are greater than 0.7. Obviously, the sensitivity and specificity of this classifier were satisfactory.

**Figure 3:**
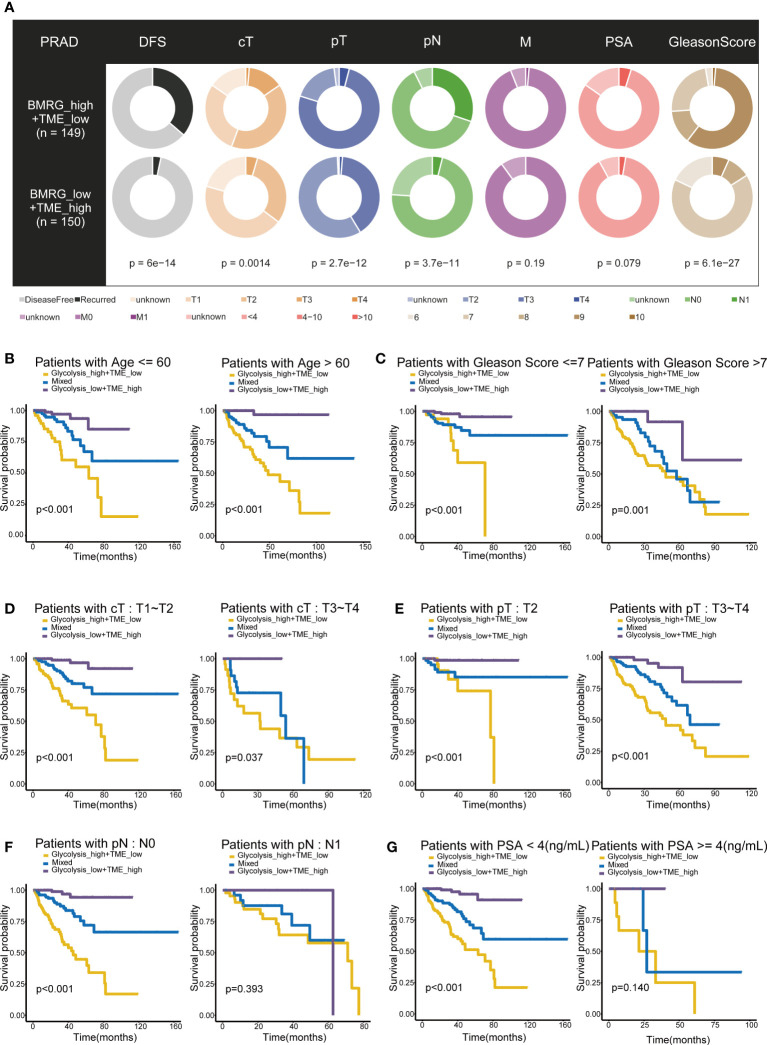
Prognostic value of Glycolysis-TME Classifier. **(A)** Different distribution of clinicopathological features of PRAD patients between the Glycolysis^high^/TME^low^ and Glycolysis^low^/TME^high^. **(B–G)** Kaplan–Meier survival subgroup analysis according to the Glycolysis-TME Classifier stratified by age, Gleason Score, clinical T stage (cT), pathological T stage (pT), pathological N stage (pN) and the concentration of prostate-specific antigen (PSA).

The two groups, Glycolysis^high^/TME^low^ and Glycolysis^low^/TME^high^, revealed significant differences in the distributions of various clinicopathological variables, including the disease-free survival, clinical T stage (cT), pathological T stage (pT), pathological N stage (pN) and Gleason Score (p< 0.05) (**Figure 3A**). Even under subgroups with different ages, Gleason Score, cT and pT stages, the differences in the survival time distributions categorized by the classifier were statistically significant (p< 0.05). Only in patients with N1 stage and prostate-specific antigen (PSA) concentrations > 4 ng/mL, the classifier performed poorly, probably owing to the sample size. These results suggested that the excellent effectiveness of the Glycolysis-TME classifier for predicting the prognosis of patients was not affected by clinicopathological factors (**Figures 3B–G**). Furthermore, univariate Cox regression analysis revealed that the cT, Gleason Score, pT, PSA and Glycolysis-TME classifier were independent predictors of DFS in patients with PRAD (p< 0.05), while cT, Gleason score and the classifier had unfavourable prognostic performance in multivariate Cox regression analysis (p< 0.05) (**Supplementary Figures 4A, B**). The clinical characteristics of the individuals in the four included cohorts were presented in **Supplementary Table 3**. In addition, we validated the prognostic capability of the Glycolysis-TME Classifier with the MSKCC cohort, GSE54460 and GSE70769 (The clinical outcome for the MSKCC cohort is DFS; the clinical outcome for GSE54460 and GSE70769 is biochemical recurrence, BCR) (**Supplementary Figures 5A–C**). Therefore, these data indicated that the Glycolysis-TME classifier was the best independent predictor of prognosis in PRAD.

### Distinct molecular signatures and mechanisms among Glycolysis-TME groups

**Figure 4:**
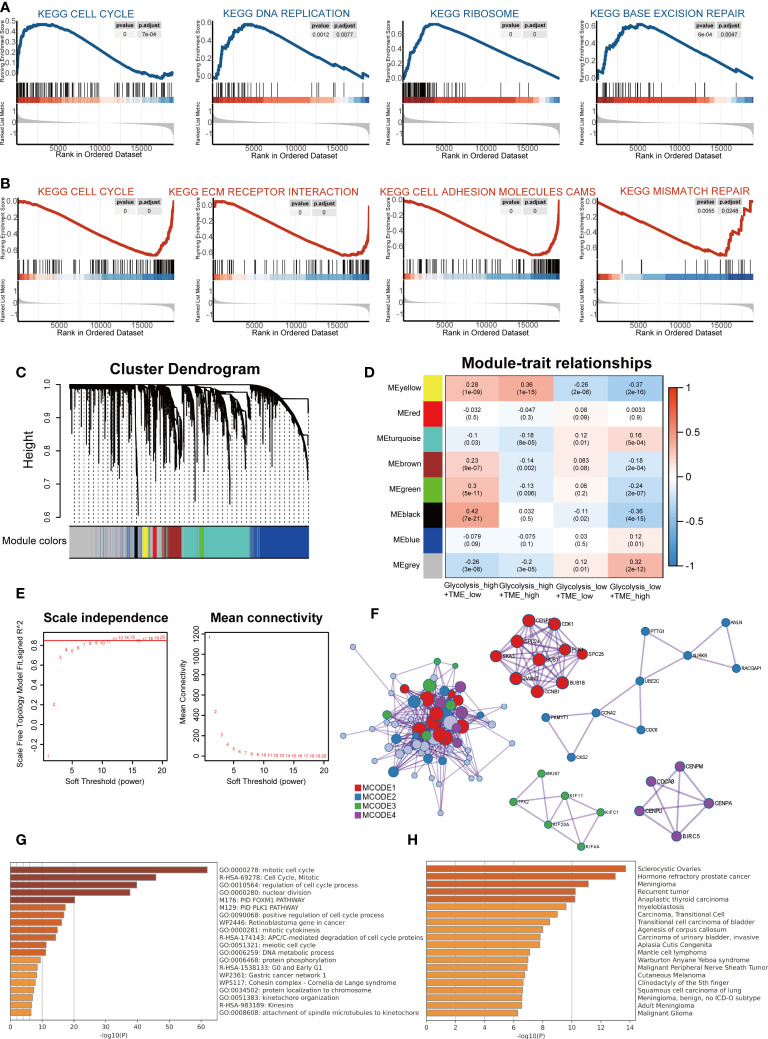
The distinct molecular functions and mechanisms among different groups. **(A)** Gene set enrichment analysis (GSEA) between groups with high- and low-glycolysis scores. **(B)** GSEA between groups with high- and low-TME scores. **(C)** Cluster Dendrogram. **(D)** Module-trait relationships. **(E)** Analysis of the network topology for various soft-thresholding powers. **(F)** Protein-protein interaction (PPI) network and four MCODEs identified in genes of the black module. **(G)** Potentially enriched terms for the gene list. **(H)** Bar graph of enriched diseases in DisGeNET.

Gene Set Enrichment Analysis confirmed differences in KEGG pathways among groups. Compared to the group with low glycolysis scores, the group with high scores was positively associated with cell cycle, DNA replication, ribosome and base excision repair, which are involved in cell proliferation and synthesis of nucleotide and protein (**Figure 4A**). When comparing the high and low TME Score groups, the results showed that the gene sets of the low TME scores group were gathered in pathways related to extracellular cell matrix (ECM) receptor interaction, cell adhesion molecules and mismatch repair and cell cycle (**Figure 4B**).

Subsequently, WGCNA was used to obtain eight modules associated with the Glycolysis-TME Classifier, and we identified the black module, which contained 67 genes most relevant to the Glycolysis^high^/TME^low^ (R = 0.42, P = 7e-21) (**Figures 4C-E**). To explore the functions of the black module genes, the Metascape portal was used for pathway analyses, and we observed that the most significant terms were related to cell proliferation, similar to the results of GSEA (**Figure 4G**). Intriguingly, in DisGeNET, these genes were significantly enriched in hormone refractory prostate cancer, second only to sclerocystic ovaries (**Figure 4H**). The Molecular Complex Detection (MCODE) algorithm divided the black module into four major MCODEs, which have been gathered and presented in **Figure 4F**. The most significant pathways in the Glycolysis^high^/TME^low^ were related to the biosynthesis of proteins and nucleic acids, whereas according to the FGSEA, there were brown fat cell differentiation and other metabolic processes displayed in the Glycolysis^low^/TME^high^ (**Supplementary Figures 6A–C**). We further performed differential gene expression analysis between the Glycolysis^high^/TME^low^ and Glycolysis^low^/TME^high^ (**Supplementary Figure 7A**), uploaded significantly different genes (p< 0.05 and log fold change, logFC > 0.5) to the online software Proteomap. It was observed that the patterns of Proteomap were quite different both in the fractions of metabolism and environmental information processing (**Supplementary Figures 7B, C**).

### Disrupted glycolysis related genes in multi-omics analysis

**Figure 5:**
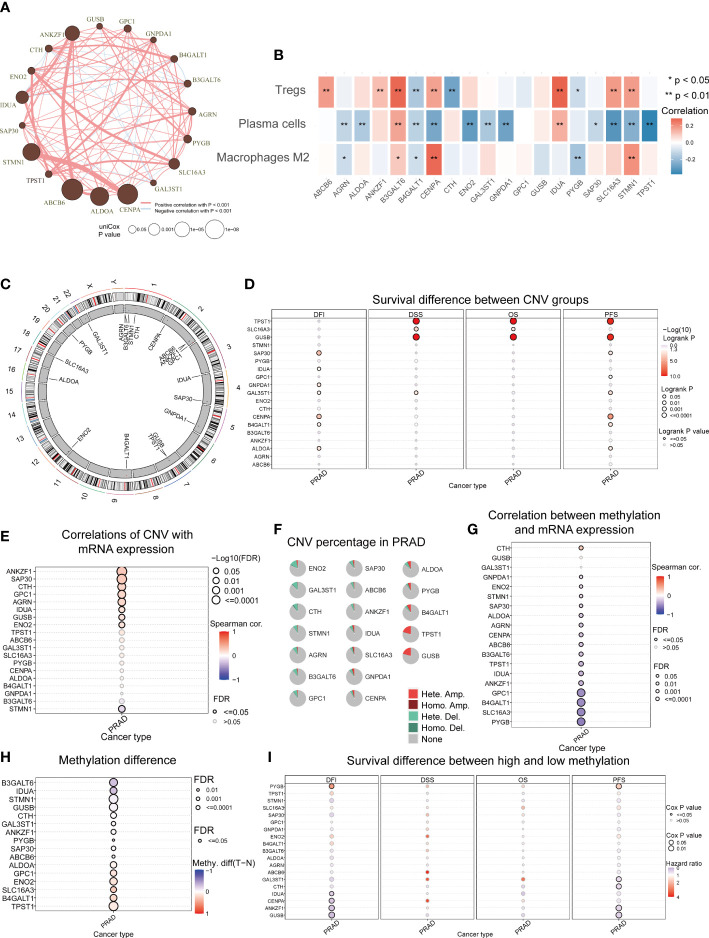
Aberrant GRGs in multi-omics analysis. **(A)** Interactions among 19 GRGs in PRAD. The circle sizes represent the P values obtained by univariate Cox regression analysis. Positive associations are marked in red, whereas negative associations are depicted in blue. **(B)** The correlation of 19 GRGs in the Glycolysis Score with the abundance of 3 TME cells in the TME Score. **(C)** The location of CNV alterations of GRGs on chromosomes. The red dots represent CNV gain while blue dots represent CNV loss. **(D)** The correlation between CNVs of GRGs and prognosis including disease-free interval (DFI), disease-specific survival (DSS), overall survival (OS) and progression-free survival (PFS). **(E)** Relationships between CNVs and mRNA expression. **(F)** Summary of CNV in GRGs. **(G)** Relationships between DNA methylation and mRNA expression. **(H)** Methylation difference between tumor and normal. **(I)** The association between methylation of GRGs and DFI, DSS, OS, PFS.

There was a comprehensive landscape of co-expression relationships among GRGs, consisting of mainly positive correlations with each other in TCGA-PRAD datasets (**Figure 5A**). We observed these GRGs exhibited primarily negative connections with plasma cells but positive correlations with the Treg (**Figure 5B**). Along with the Glycolysis Score increased, M2 and Treg also presented an upward trend, while plasma cells decreased (**Supplementary Figures 8A–C**). The chromosomal location of CNV alteration for each gene was shown in **Figure 5C**. Furthermore, **Figures 5E, G** confirmed the association between two important factors and expression levels of GRGs. As demonstrated above, the relationship between CNVs, methylation levels and mRNA levels were mainly positively and negatively correlated, respectively. In addition, for some GRGs, two factors can affect the prognosis (**Figures 5D, I**). Differential DNA methylation patterns were explored, TPST1, B4GALT1, SLC16A3, ENO2, GPC1, and ALDOA were then found to be hypermethylated in PRAD (**Figure 5H**). Meanwhile, a global profile depicted the constitute of Heterozygous/Homozygous CNV of GRGs in **Figure 5F**. Among the cancer-related signaling pathways, GRGs had high levels of activation in epithelial-mesenchymal transition (EMT), hormone androgen receptor (AR) and cell cycle signaling pathway (**Supplementary Figure 9**).

**Figure 6:**
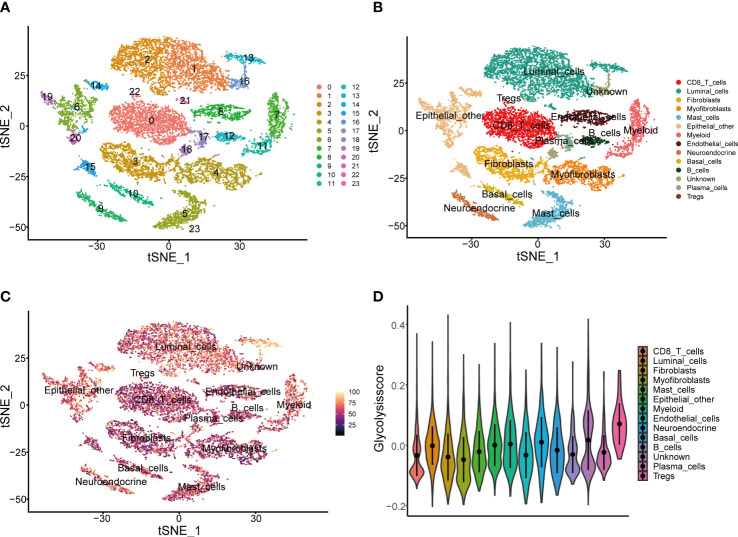
Presentation of Glycolysis Score at the single-cell level. **(A, B)** Single-cell analysis based on GSE137829. **(C)** t-SNE plot of single cells colored by the Glycolysis Score. **(D)** The comparison of Glycolysis Score among different cell types.

To further validate the Glycolysis Score at the single-cell level, we initially performed quality control to filter cells from two CRPC samples in GSE137829 (**Supplementary Figures 10A–C**). The top 3000 variable genes were marked with red dots, while the 10 with the largest standard deviation were labeled (**Supplementary Figure 11A**). The expression values of markers for epithelial cells, immune cells, other cells and GRGs in different clusters were visualized (**Supplementary Figures 11B–E**). We next applied the t-distributed stochastic neighbor embedding (t-SNE) algorithm to display the single-cell data, and successfully classified cells into 24 clusters (**Figure 6A**), which were ultimately annotated as CD8^+^ T, fibroblasts, myofibroblasts, mast, myeloid, endothelial, neuroendocrine, luminal, basal, other epithelial, plasma, regulatory T, B and unknown cells (**Figure 6B**). The composite expression score of the 19 genes in the Glycolysis Score, was highest in Tregs (**Figures 6C, D**).

### Profiles of tumor somatic mutations among groups

**Figure 7:**
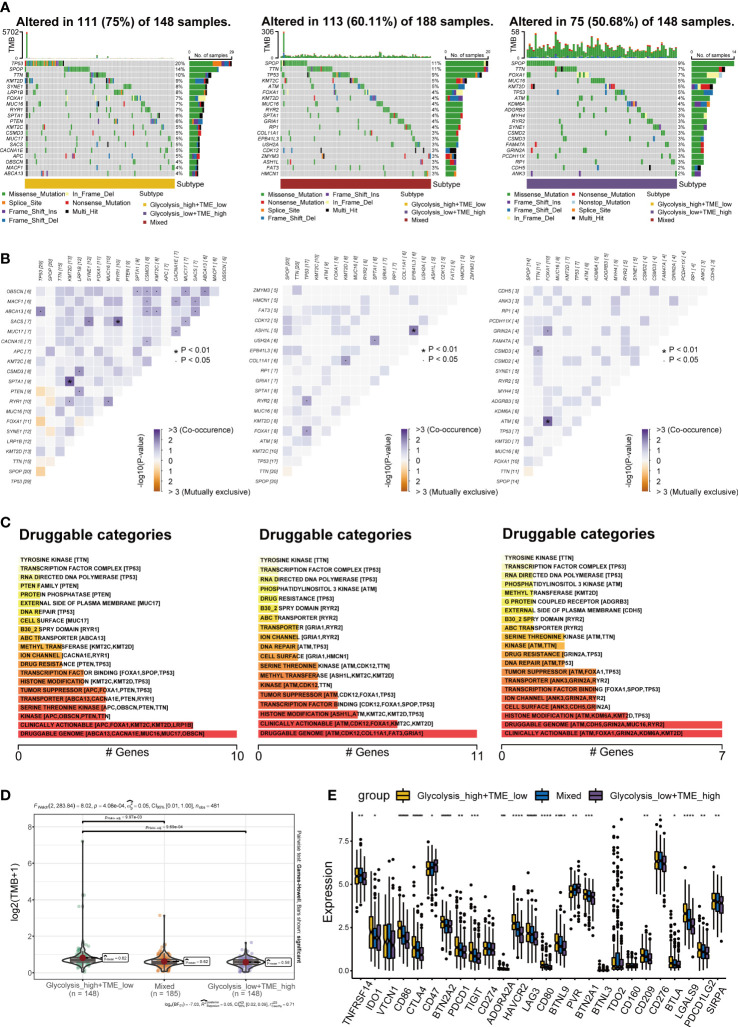
Landscape of somatic mutations **(A)** Oncoplots exhibiting the mutation patterns with the top 20 most frequently mutated genes among the Glycolysis^high^/TME^low^, Mixed group and Glycolysis^low^/TME^high^. **(B)** The co-occurrence and mutually exclusive among high-frequency mutated genes. **(C)** Bar plots showing potential druggable categories. **(D, E)** Differences in tumor mutational burden (TMB) and classical immune checkpoints among separate groups. *p< 0.05; **p< 0.01; ***p< 0.001; ****p< 0.0001.

The distribution of somatic mutations among three groups was displayed with the oncoplot, and the top 20 variant mutations were identified in each group. The frequency of mutations and co-occurrence was higher in the Glycolysis^high^/TME^low^ (**Figures 7A, B**), and higher frequency mutations in the Glycolysis^high^/TME^low^ include TP53, SPOP and KMT2D. Moreover, we explored potential therapeutic targets according to the mutation data. In three distinct groups, druggable genes were classified into 21, 19, and 21 categories, respectively, including druggable genome, clinically actionable and histone modification (**Figure 7C**). As a predictor of immunotherapy sensitivity, a higher TMB score was observed in the Glycolysis^high^/TME^low^ than in the other two groups (**Figure 7D**). In addition, most of the immune checkpoint expression values in this group were the highest among all groups (**Figure 7E**). Following the above analysis, it is reasonable to speculate that Glycolysis^high^/TME^low^ is more suitable for immunotherapy.

### Glycolysis-TME Classifier predicted drug sensitivity

**Figure 8:**
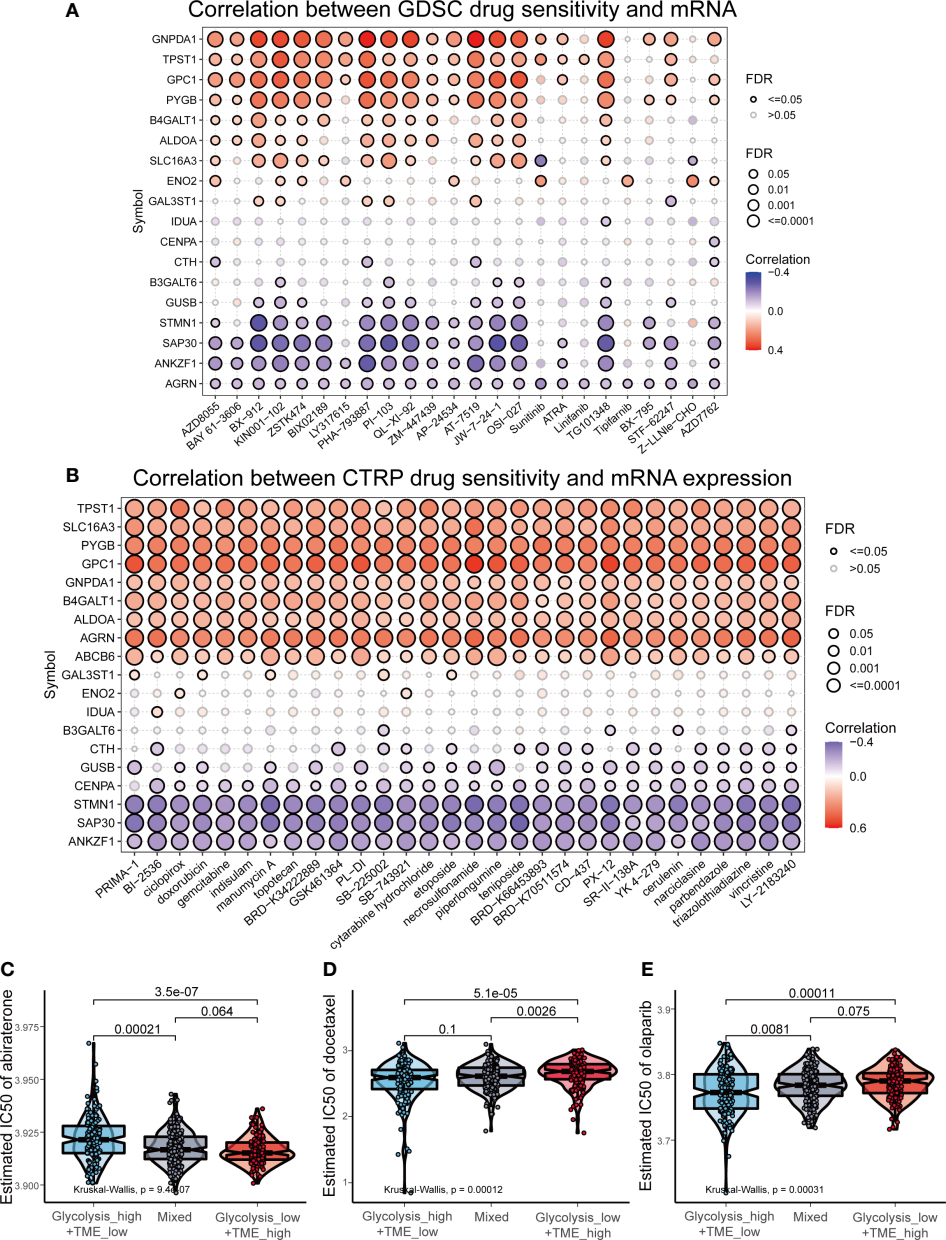
Drug sensitivity analysis in prostate cancer. **(A, B)** The correlation between drug sensitivity and expression of GRGs based on Genomics of Drug Sensitivity in Cancer (GDSC) and Cancer Therapeutics Response Portal (CTRP) datasets. **(C-E)** The comparison of the estimated IC50 of abiraterone, docetaxel and olaparib.

Given the mechanisms underlying the differences among different groups, we investigated the association between drug sensitivity and expression of GRGs in prostate cancer based on the GDSC and CTRP databases, and observed that GNPDA1, TPST1, GPC1, PYGB, B4GALT1 and ALDOA were positively correlated with the IC50 of most drugs, while ANKZF1, SAP30 and STMN1 were mainly negatively correlated (**Figures 8A, B**). When comparing the IC50 of commonly used clinical drugs for PCa among the three groups, the results revealed that patients in the Glycolysis^low^/TME^high^ are probably more sensitive to abiraterone (**Figure 8C**), a selective inhibitor of 17α-hydroxylase and 17,20-lyase (34). Whereas, in terms of chemotherapy (docetaxel) and PARP inhibitors (olaparib), the Glycolysis^high^/TME^low^ displayed the lowest IC50 values (**Figures 8D, E**).

## Discussion

Prostate cancer is characterized by high heterogeneity from the clinical, morphological and molecular perspectives (35). In this regard, TME heterogeneity has profound effects on tumor energy metabolism, particularly the glycolytic process (36). Growing evidence has indicated that glycolysis activity, as the hallmark of tumors and a convergent property, is activated by different oncogenic drivers or hypoxia (37), which suppresses immune response. Considering the complexity of TME under metabolic reprogramming affecting PCa progression and the bulk of researches that have focused on either a specific regulatory GRG or some type of immune cell, it is necessary to comprehensively establish a prognostic classifier that incorporates both. Despite being an inert tumor that possesses a high overall survival (OS) rate, PCa progresses rapidly after the turning point of recurrence, even with the available clinical interventions (8). Thus, consistent with other PCa prognostic studies (38–42), DFS was selected as the endpoint of this study.

In this study, we identified DEGs from the Hallmark glycolysis gene set primarily enriched in carbon metabolism but also amino acid and nucleotide biosynthesis. This is consistent with the current understanding of *in vivo* tumor metabolism, which indicates that different metabolic pathways have multiple interconnections in various ways to produce ATP, nucleotides and proteins for cell proliferation (43). With further investigation into prognosis-related GRGs and LASSO analysis to reduce the number of GRGs, we obtained 19 key genes. Some of these GRGs have been previously reported to involve in glycolysis PCa development. Among them, the decrease in the enzyme encoding gene α-L-iduronidase (IDUA) is accompanied an increase glycolytic flux, due to the increased expression of glycolytic enzymes (44). Enolase 2 (ENO2), a recognized tumor biomarker for PCa, is responsible for converting 2-phosphoglycerate into phosphoenolpyruvate during the glycolytic process (45). In addition, overexpression of centromere protein A (CENPA), a regulator of metabolic reprogramming, may restore growth and glycolysis in tumor cells (46). According to Xie et al. (47), the expression of solute carrier family 16 member 3 (SLC16A3), a crucial lactate transporter in glycolysis, was positively correlated with worse DFS and Gleason Score in prostate cancer. Furthermore, it has been reported that phospho-ALDOA facilitates metabolic reprogramming and cell proliferation (48). Lactate depletion and tumor growth are inhibited by ALDOA knockdown (49). A study by Zhang et al. also reported that Tm cells mainly expressed the brain form of PYG (PYGB), which improves the quality and activity in intracellular glycogenolysis for ensuring the rapid recall response of Tm (50). Interestingly, these genes found in the glycolysis-related signature are predominantly associated with the androgen receptor. It is prominent that PCa is an androgen-dependent tumor, and AR is essential for the pathogenesis of PCa (51). In anabolic regulation, AR directly elevates key enzymes involved in glycolysis, including hexokinase I and II (HK1 and HK2) (52), suggesting that AR might also regulate the glycolysis-related signature identified in this study. There are certainly further facets of how these genes impact glycolysis in TME of PCa, that should further be investigated to provide new insights into the disease etiology.

The three immune cells with specific immunophenotypes in the TME Score have crucial impacts on PCa tumorigenesis, progression and metastasis through direct interactions with tumor cells or indirect cytokine release (53), and their roles in the dynamic glycolytic metabolism of tumors have recently been a focus. Notably, Treg, which had the highest Glycolysis Score in the single-cell analysis, maintains its immunosuppressive and proliferative capacity through utilizing lactic acid, a by-product of glycolytic metabolism, produced by tumor cells in the TME (54). Lactate drives the polarization of tumor-associated macrophages (TAM), characterized by elevated expression of vascular endothelial growth factor (VEFG), and differentiation into M2 macrophages (55). These indicate that an acidic environment with high glycolysis flux can effectively activate Treg and M2, and both of which have been reported to be poor prognostic factors inhibiting anti-tumor immunity in previous studies (56, 57). Conversely, the log-rank test revealed that the plasma cell was associated with longer DFS, which is consistent with a report on PCa by Adam et al. (58). In general, it is clear that the role of the glycolytic switch is crucial for the anti-tumor immune response (59).

Based on the Glycolysis Score and the TME Score, the Glycolysis-TME classifier was created. GSEA and WGCNA revealed that Glycolysis^high^/TME^low^ was activated in the cell cycle, DNA replication, mismatch repair, and base excision repair pathway, all of which are necessary for cancer etiology. In FSGEA, it was observed that brown fat cell differentiation was elevated in the Glycolysis^low^/TME^high^, and brown fat activated by cold promotes the uptake of glucose uptake, thus, significantly inhibiting tumor growth (60). In addition to cell adhesion molecules involved in tumor metastasis, the MAPK signaling and PI3K-Akt signaling pathways were more enriched in the Glycolysis^high^/TME^low^ than in the Glycolysis^low^/TME^high^, as confirmed by Proteomaps. In addition, there were notable differences in clinicopathological characteristics among the groups. These factors, as previously mentioned, may explain why the Glycolysis^high^/TME^low^ exhibited a worse prognosis than the other two groups, providing a potential mechanism underlying the prognostic power of the classifier.

Immunotherapy has become an important treatment modality for cancer in recent years. However, owing to the characteristic of prostate cancer as a cold tumor, its immunotherapy response is not ideal. However, there are still some people for whom immunotherapy is effective (61). Therefore, optimizing immunotherapy and predicting individual immune responses more accurately have emerged as research hotspots. We discovered that the expression of most immune checkpoints was highest in the Glycolysis^high^/TME^low^, and the same was observed in the comparison of TMB. Occasionally, higher TMB has more neoantigens that the immune system can recognize and is an indicator of immunotherapy sensitivity (62). These results indicate that the Glycolysis^high^/TME^low^ is more sensitive to immune checkpoint inhibitors (ICIs). Chang et al. reported that blocking PD-L1 can impair tumor glycolysis by decreasing the expression of glycolytic enzymes (63). In contrast, lactate at the high glycolytic level stimulates PD-1 expression in Treg cells (64). Hence, clinically targeting the glycolysis metabolism directly inhibits the growth and proliferation of tumor cells, thus improving the efficacy of immunotherapy (65, 66). Further research is necessary since it can be believed that the combination of ICIs and medications that target glycolysis may shape the future of PCa treatment, which warrants further investigation. We finally predicted the sensitivity of various groups to antiandrogens, chemotherapeutic agents, and targeted drugs to further improve the value of the classifier in clinical application.

There are still some limitations in our study. First, the bulk RNA-seq and microarray data were prominently obtained from public databases, which might have other unknown implications, such as prostatitis (67). Second, the population primarily consisted of Whites and Blacks. Additional real-world studies including Asians or other races are needed to validate the effectiveness of the classifier. Therefore, we plan to apply this classifier in our hospital to demonstrate its clinical value in a Chinese population-based cohort. Conclusively, we innovatively constructed the Glycolysis-TME Classifier, with TCGA-PRAD as the training set and MSKCC cohort, GSE54460 and GSE70769 as the validation sets. Due to the adequate prognostic capacity for PCa patients and guidance on the use of medicines, it might be an effective tool for future clinical management.

## Data availability statement

The datasets presented in this study can be found in online repositories. The names of the repository/repositories and accession number(s) can be found in the article/**Supplementary Material**.

## Author contributions

TG, JW and SY contributed equally to this work. Conceptualization: TG, SR and YH. Investigation: TG, JW and SY. Methodology, software, visualization, formal analysis and data curation: TG, JW, SY, XM, XZ and SX. Writing—original draft: TG, XM, SR and YH. Writing - review & editing: TG, SR and YH. All authors contributed to the article and approved the submitted version.

## Funding

This work was supported by two grants from the Key Research and Development Program of Jiangsu Province (No. BE2020654 and No. BE2020655) and a grant from the General Program of Jiangsu Health Commission (No. H2019040).

## Conflict of interest

The authors declare that the research was conducted in the absence of any commercial or financial relationships that could be construed as a potential conflict of interest.

## Publisher’s note

All claims expressed in this article are solely those of the authors and do not necessarily represent those of their affiliated organizations, or those of the publisher, the editors and the reviewers. Any product that may be evaluated in this article, or claim that may be made by its manufacturer, is not guaranteed or endorsed by the publisher.
