# Supplementary material for: A combined signature of glycolysis and immune landscape predicts prognosis and therapeutic response in prostate cancer
Source: Front Endocrinol (Lausanne). 2022 Oct 21;13:1037099. doi: 10.3389/fendo.2022.1037099 (PMC9634133; doi:10.3389/fendo.2022.1037099)
Supplement: Supplementary file 1 [file DataSheet_1.docx]

**Supplementary Table 1** | Construction of Glycolysis Score.

| **Genes** | **Coef** | **Bootstrap (SD)** | **Coef/ Bootstrap (SD)** |
| --- | --- | --- | --- |
| B3GALT6 | 0.149534 | 0.325506 | 0.45939 |
| IDUA | 0.255754 | 0.300411 | 0.851345 |
| ANKZF1 | 0.351852 | 0.365576 | 0.962461 |
| ENO2 | 0.003861 | 0.218066 | 0.017708 |
| CENPA | 0.411486 | 0.248455 | 1.656183 |
| ABCB6 | -0.06507 | 0.79212 | -0.08215 |
| GUSB | -0.4391 | 0.261407 | -1.67977 |
| SLC16A3 | 0.248912 | 0.254555 | 0.977831 |
| SAP30 | 0.40578 | 0.381459 | 1.063757 |
| GPC1 | 0.196373 | 0.274067 | 0.716516 |
| ALDOA | 0.26254 | 0.278525 | 0.942611 |
| PYGB | -0.37713 | 0.256472 | -1.47047 |
| B4GALT1 | -0.52958 | 0.286706 | -1.84713 |
| GAL3ST1 | 0.097425 | 0.224336 | 0.434282 |
| AGRN | 0.303603 | 0.277383 | 1.094526 |
| TPST1 | 0.264814 | 0.302818 | 0.874499 |
| GNPDA1 | 0.387684 | 0.363865 | 1.06546 |
| CTH | -0.40181 | 0.292374 | -1.37431 |
| STMN1 | -0.11057 | 0.299294 | -0.36944 |

Coef: coefficients of multivariate regression; Bootstrap (SD): standard deviations computed with bootstrap.

**Supplementary Table 2** | Construction of TME Score.

| **Immune Cells** | **Coef** | **Bootstrap (SD)** | **Coef/ Bootstrap (SD)** |
| --- | --- | --- | --- |
| Macrophages M2 | 5.512312 | 1.614788 | 3.413644 |
| Plasma cells | -2.99847 | 1.556063 | -1.92696 |
| T cells regulatory (Tregs) | 7.780528 | 3.308667 | 2.35156 |

Coef: coefficients of multivariate regression; Bootstrap (SD): standard deviations computed with bootstrap.

**Supplementary Table 3** | Clinicopathological features of patients in TCGA-PRAD cohort, MSKCC cohort, GSE54460 and GSE70769.

|  | **TCGA (N=491)** | **MSKCC (N=140)** | **GSE54460 (N=100)** | **GSE70769 (N=92)** | **Total (N=823)** |
| --- | --- | --- | --- | --- | --- |
| **Age** |  |  |  |  |  |
| <=60 | 221 (45.0%) | 0 (0%) | 44 (44.0%) | 0 (0%) | 265 (32.2%) |
| >60 | 270 (55.0%) | 0 (0%) | 56 (56.0%) | 0 (0%) | 326 (39.6%) |
| unknown | 0 (0%) | 140 (100%) | 0 (0%) | 92 (100%) | 232 (28.2%) |
| **cT** |  |  |  |  |  |
| T1~T2 | 347 (70.7%) | 133 (95.0%) | 81 (81.0%) | 80 (87.0%) | 641 (77.9%) |
| T3~T4 | 53 (10.8%) | 6 (4.3%) | 18 (18.0%) | 9 (9.8%) | 86 (10.4%) |
| unknown | 91 (18.5%) | 1 (0.7%) | 1 (1.0%) | 3 (3.3%) | 96 (11.7%) |
| **pT** |  |  |  |  |  |
| T0 | 0 (0%) | 0 (0%) | 0 (0%) | 1 (1.1%) | 1 (0.1%) |
| T1~T2 | 185 (37.7%) | 86 (61.4%) | 81 (81.0%) | 47 (51.1%) | 399 (48.5%) |
| T3~T4 | 300 (61.1%) | 54 (38.6%) | 18 (18.0%) | 42 (45.7%) | 414 (50.3%) |
| unknown | 6 (1.2%) | 0 (0%) | 1 (1.0%) | 2 (2.2%) | 9 (1.1%) |
| **pN** |  |  |  |  |  |
| N0 | 341 (69.5%) | 0 (0%) | 0 (0%) | 0 (0%) | 341 (41.4%) |
| N1 | 78 (15.9%) | 0 (0%) | 0 (0%) | 0 (0%) | 78 (9.5%) |
| unknown | 72 (14.7%) | 140 (100%) | 100 (100%) | 92 (100%) | 404 (49.1%) |
| **M** |  |  |  |  |  |
| M0 | 450 (91.6%) | 0 (0%) | 0 (0%) | 0 (0%) | 450 (54.7%) |
| M1 | 2 (0.4%) | 0 (0%) | 0 (0%) | 0 (0%) | 2 (0.2%) |
| unknown | 39 (7.9%) | 140 (100%) | 100 (100%) | 92 (100%) | 371 (45.1%) |
| **PSA** |  |  |  |  |  |
| <4 | 408 (83.1%) | 0 (0%) | 9 (9.0%) | 7 (7.6%) | 424 (51.5%) |
| 4~10 | 11 (2.2%) | 0 (0%) | 58 (58.0%) | 55 (59.8%) | 124 (15.1%) |
| >10 | 15 (3.1%) | 0 (0%) | 30 (30.0%) | 28 (30.4%) | 73 (8.9%) |
| unknown | 57 (11.6%) | 140 (100%) | 3 (3.0%) | 2 (2.2%) | 202 (24.5%) |
| **GleasonScore** |  |  |  |  |  |
| <=7 | 290 (59.1%) | 117 (83.6%) | 86 (86.0%) | 75 (81.5%) | 568 (69.0%) |
| >7 | 201 (40.9%) | 21 (15.0%) | 14 (14.0%) | 15 (16.3%) | 251 (30.5%) |
| unknown | 0 (0%) | 2 (1.4%) | 0 (0%) | 2 (2.2%) | 4 (0.5%) |

cT: clinical T stage; pT: pathological T stage; pN: pathological N stage; M: metastasis stage; PSA: prostate-specific antigen.

**
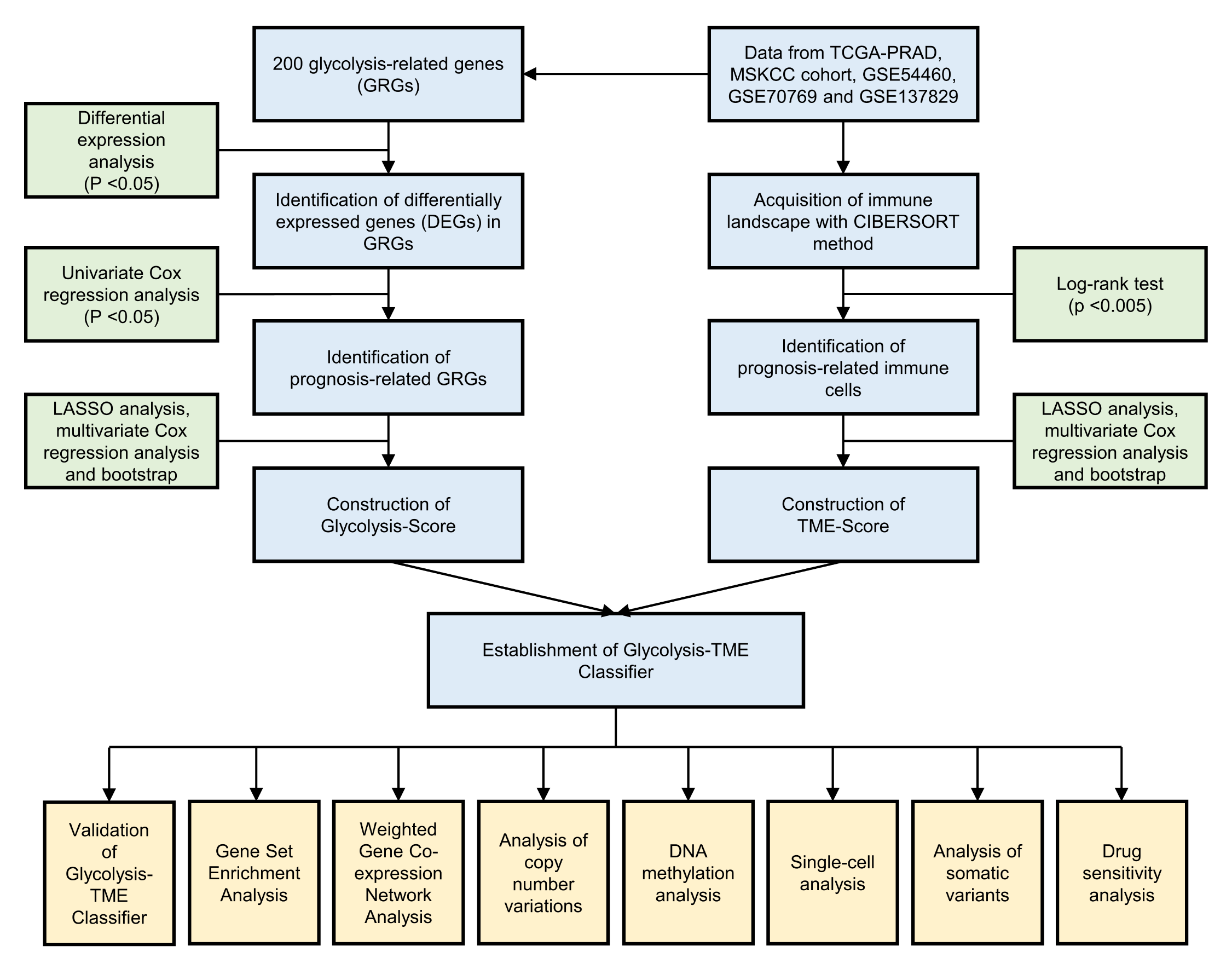
**

**Supplementary Figure 1** | The simplified workflow of this study.

**
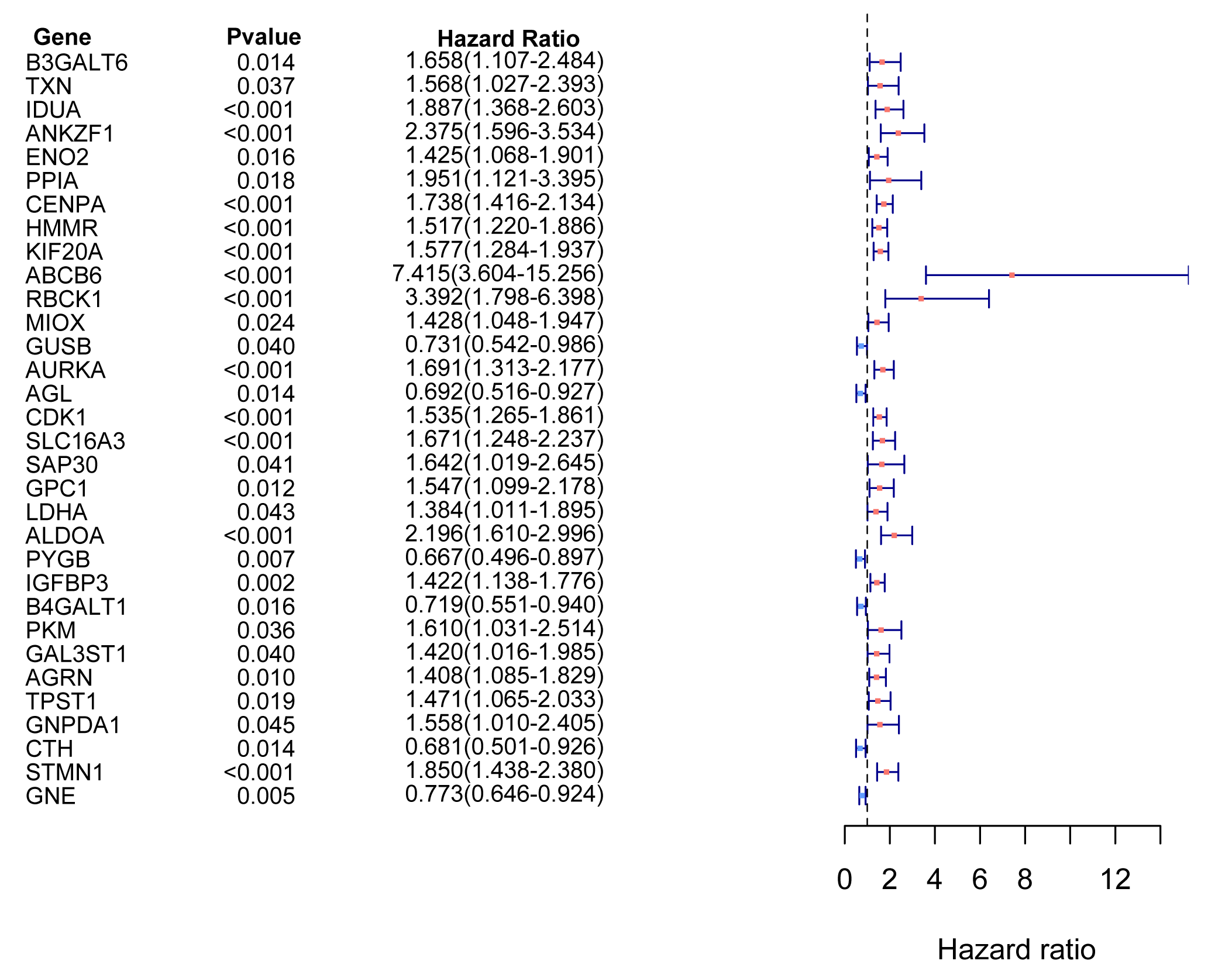
**

**Supplementary Figure 2** | Univariate Cox regression analysis revealing 32 DFS-related GRGs.

**
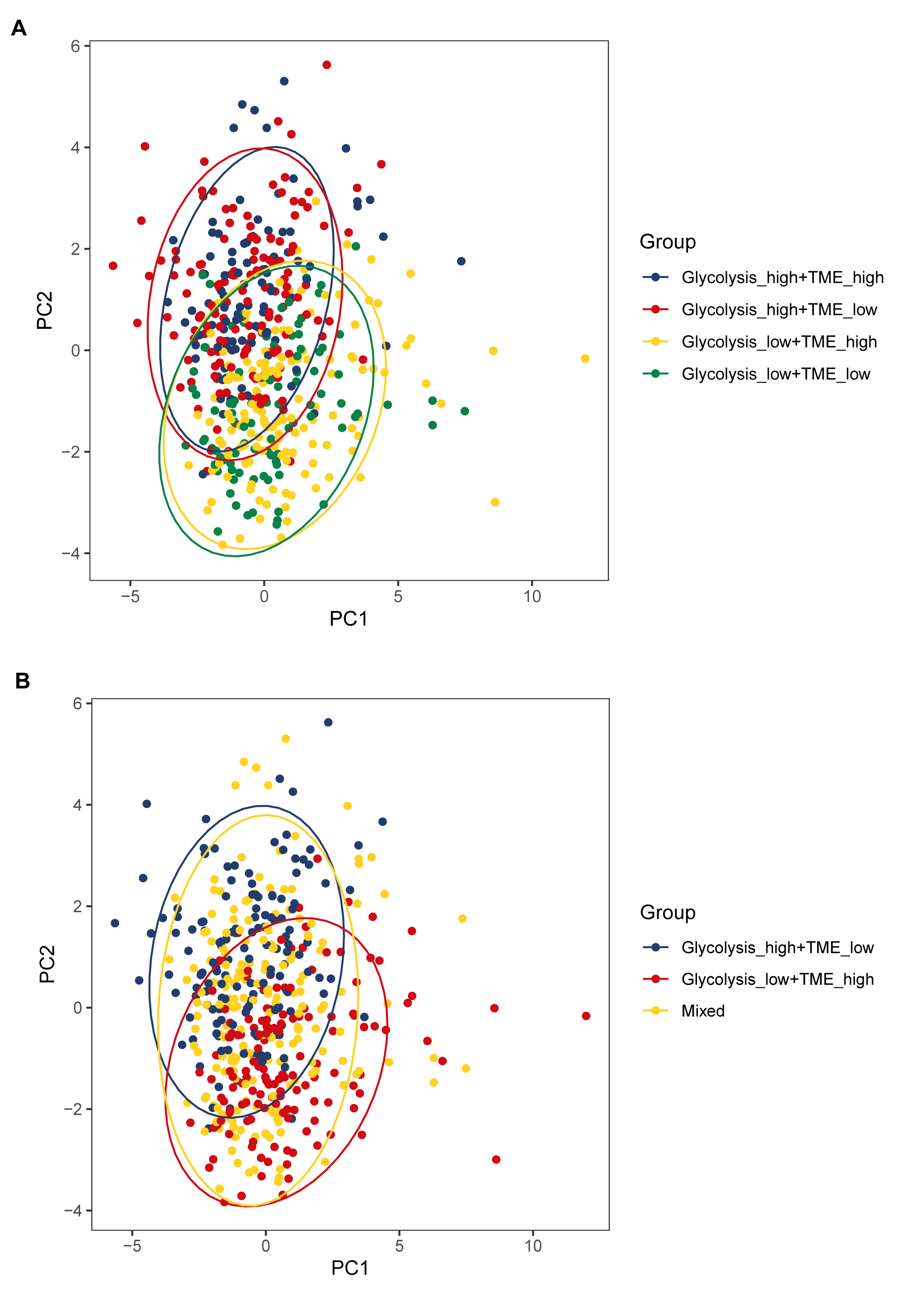
**

**Supplementary Figure 3** | Distribution of varying groups in TCGA-PRAD. **(A, B)** Principal component analysis (PCA) of PCa samples by the expression of 19 GRGs in the Glycolysis Score. Different colors indicate different groups.

**
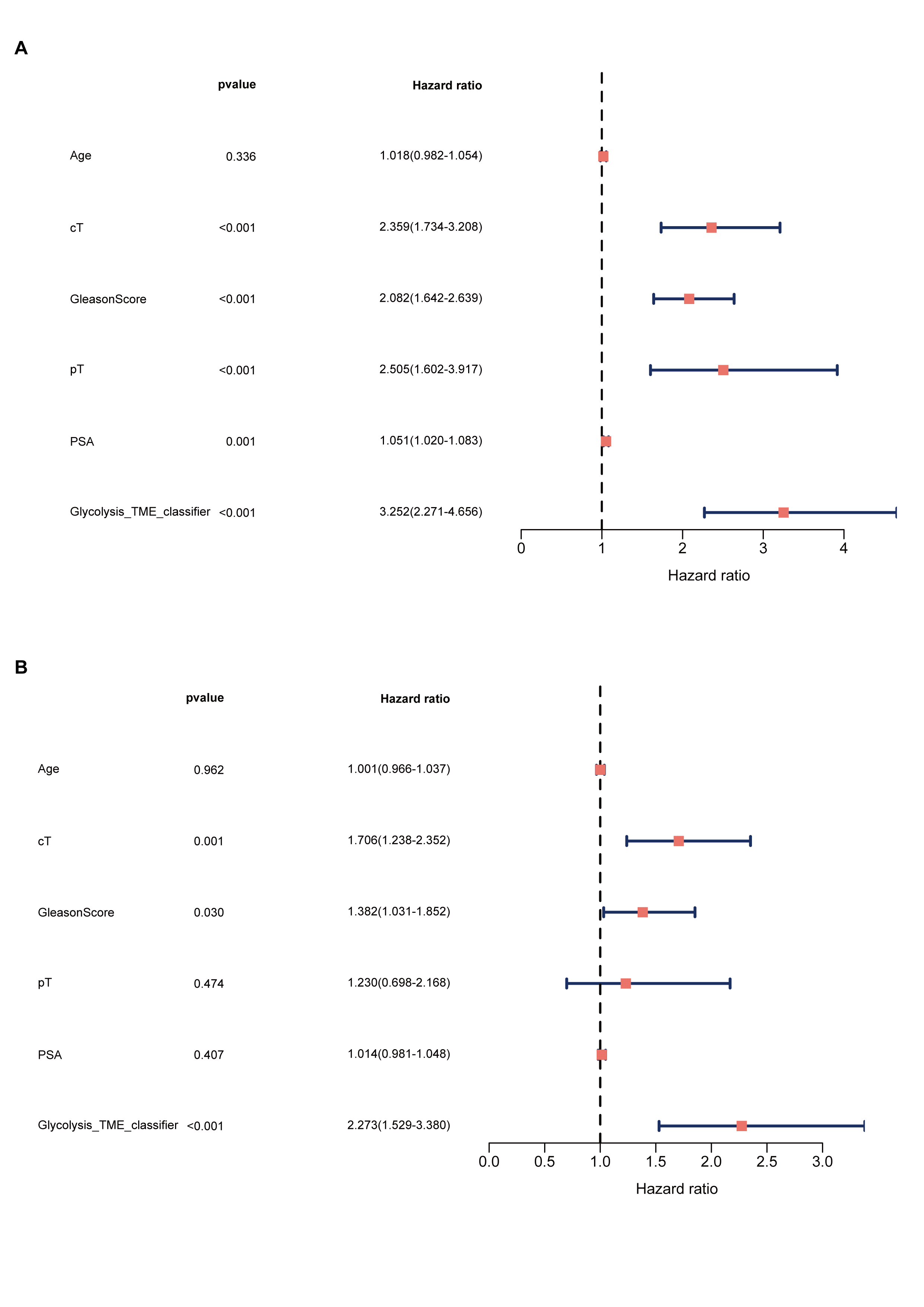
**

**Supplementary Figure 4** | Analysis of independent prognostic factors. **(A, B)** Univariate and multivariate Cox regression analysis of age, cT, Gleason Score, pT, PSA and the Glycolysis-TME Classifier.

**
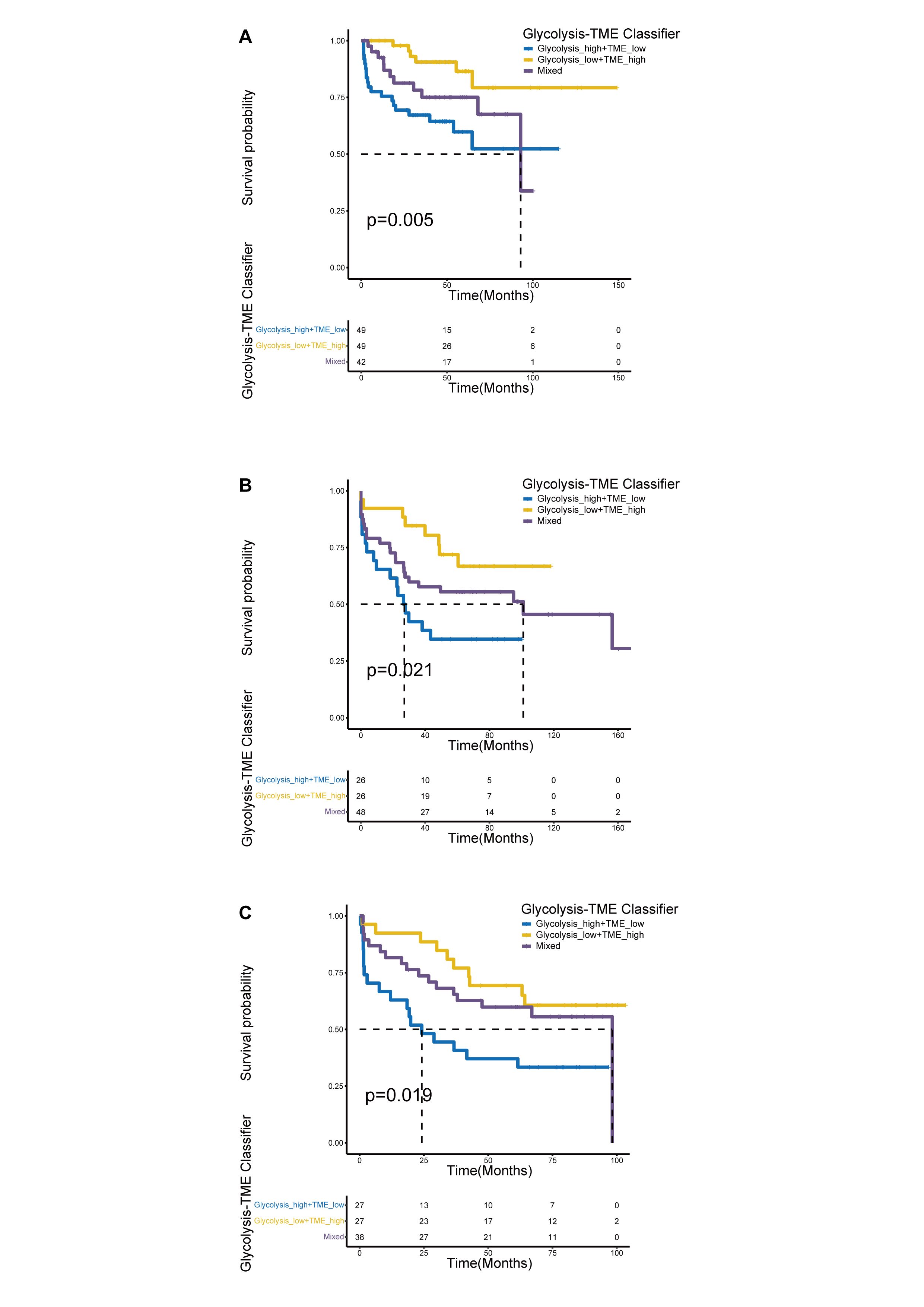
**

**Supplementary Figure 5** | Validation of Glycolysis-TME Classifier. **(A)** Kaplan–Meier analysis for DFS of patients from the MSKCC cohort between groups defined by the Glycolysis-TME Classifier. **(B)** Kaplan–Meier analysis for biochemical recurrence (BCR) of patients from GSE54460. **(C)** Kaplan–Meier analysis for BCR of patients from GSE70769.

**
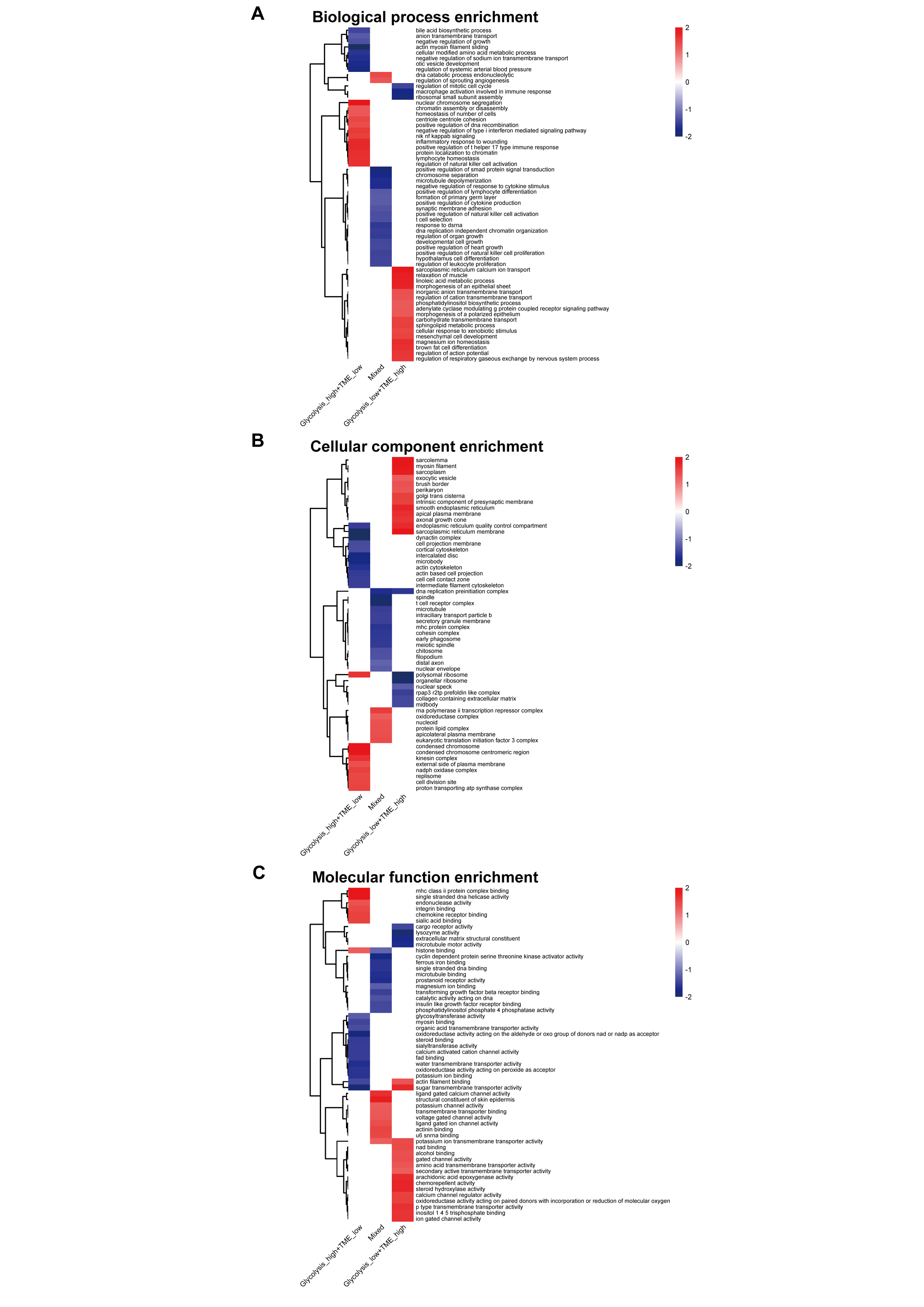
**

**Supplementary Figure 6** | Fast gene set enrichment analysis. **(A-C)** Distinct patterns of biological process (BP), cellular component (CC) and molecular function (MF) enrichment.

**
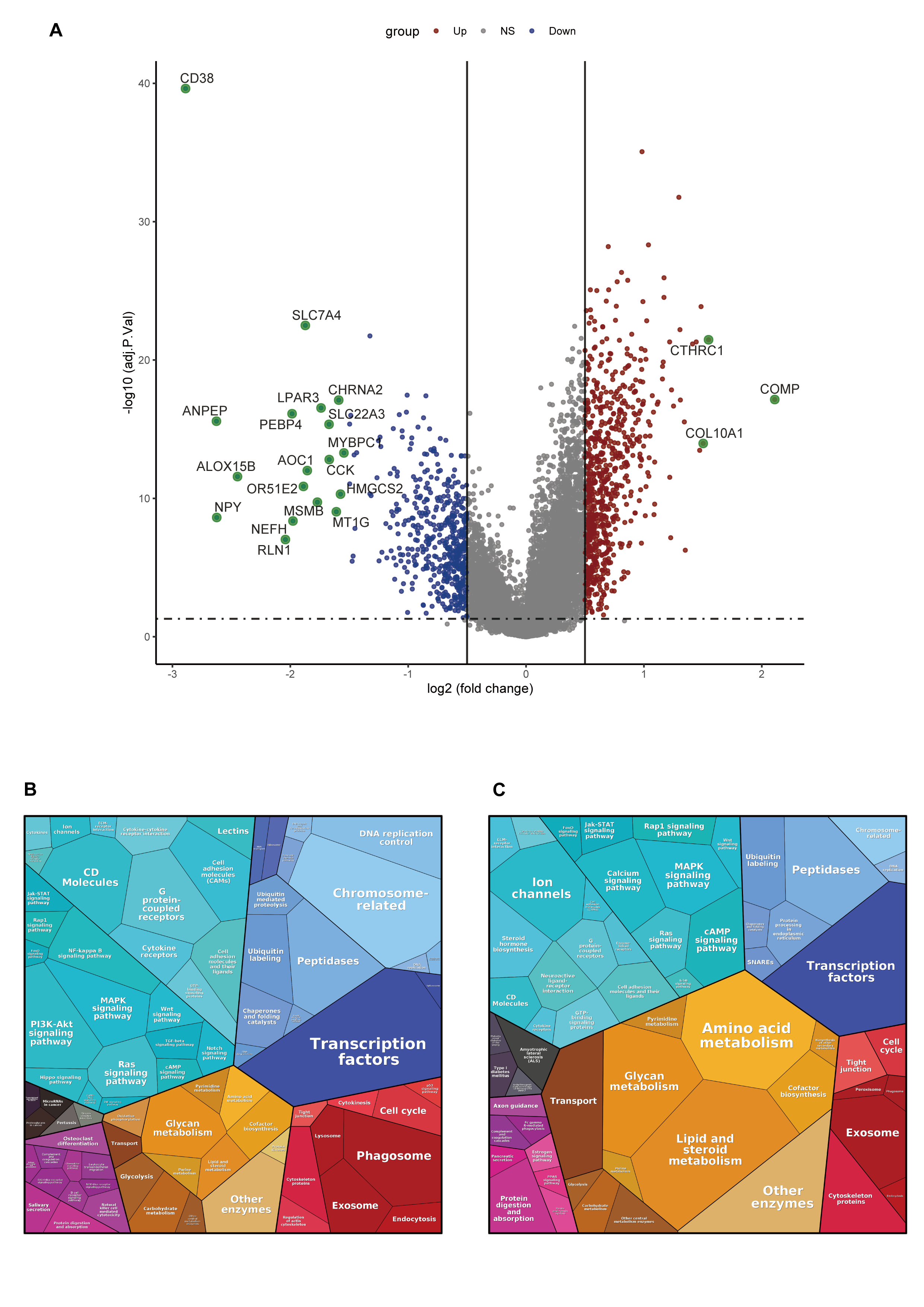
**

**Supplementary Figure 7** | Functional analysis showing different KEGG pathway profiles. **(A)** Volcano plot of DEGs between the Glycolysis^high^/TME^low^ and Glycolysis^low^/TME^high^. **(B)** The proteomap pattern of Glycolysis^high^/TME^low^. **(C)** The proteomap pattern of Glycolysis^low^/TME^high^.

**
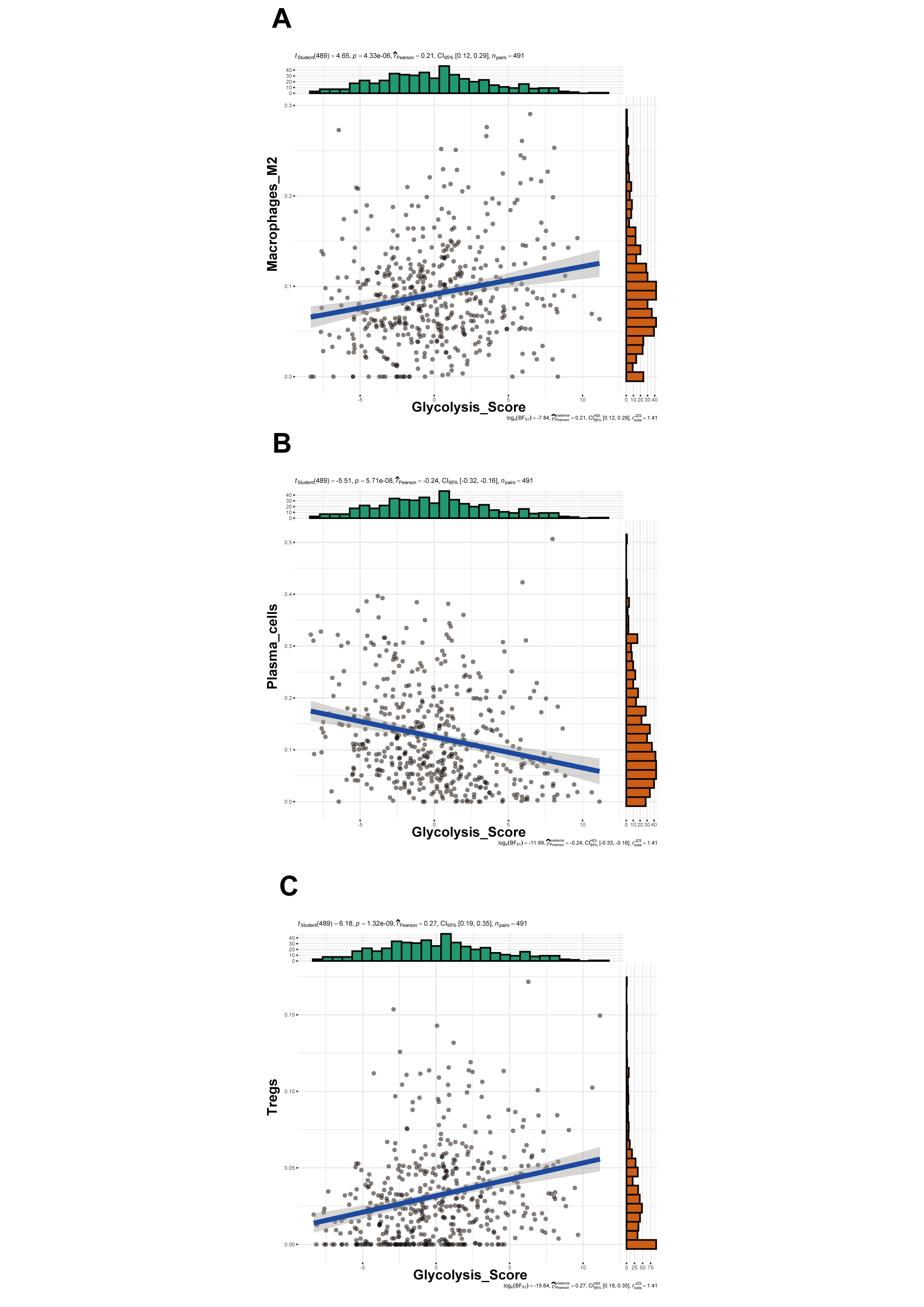
**

**Supplementary Figure 8** | The correlation between the abundance of **(A)** M2 macrophages, **(B)** plasma cells, **(C)** Tregs and the Glycolysis Score.

**
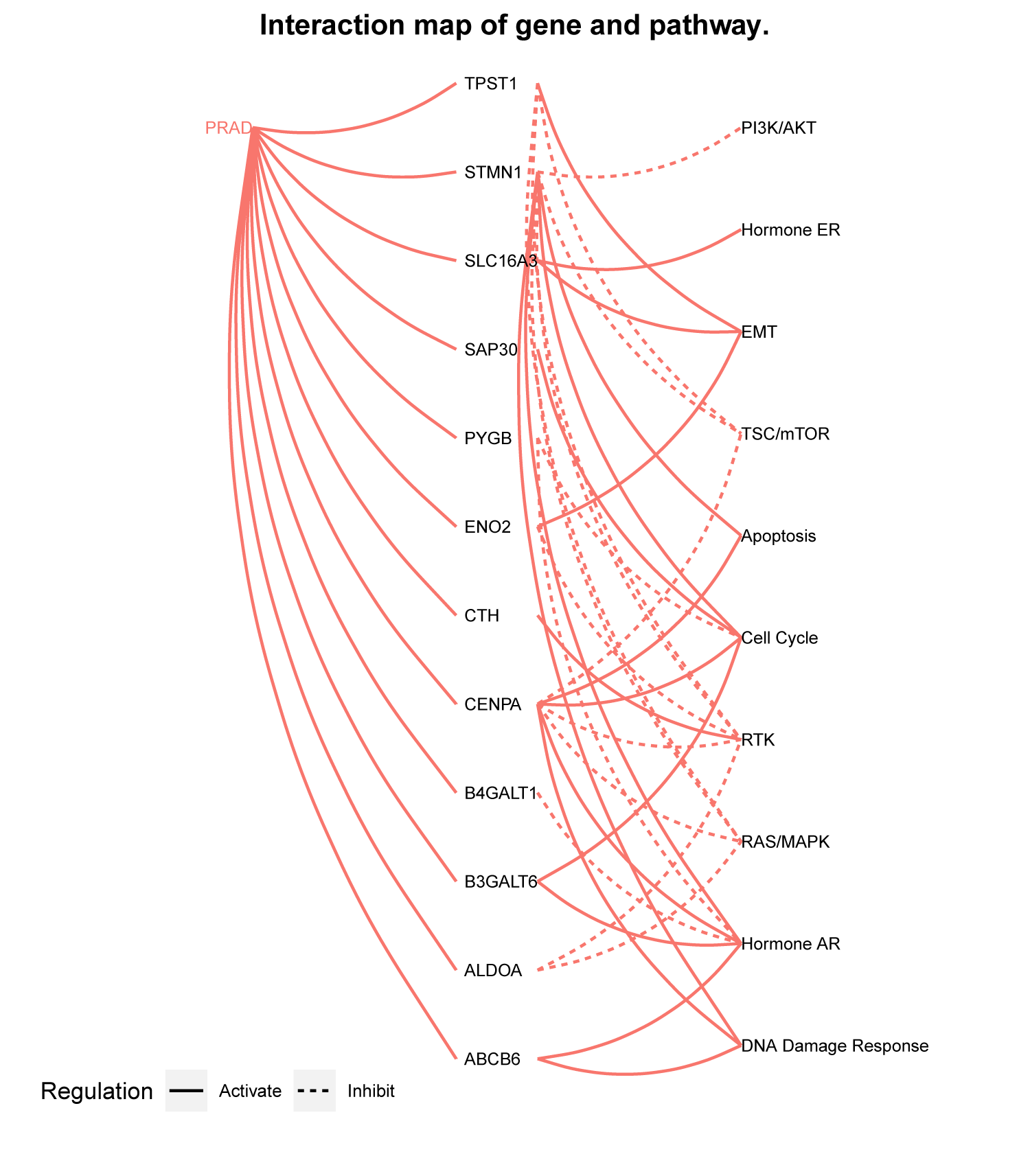
**

**Supplementary Figure 9** | The interaction of GRGs and important signaling pathways in cancer.

**
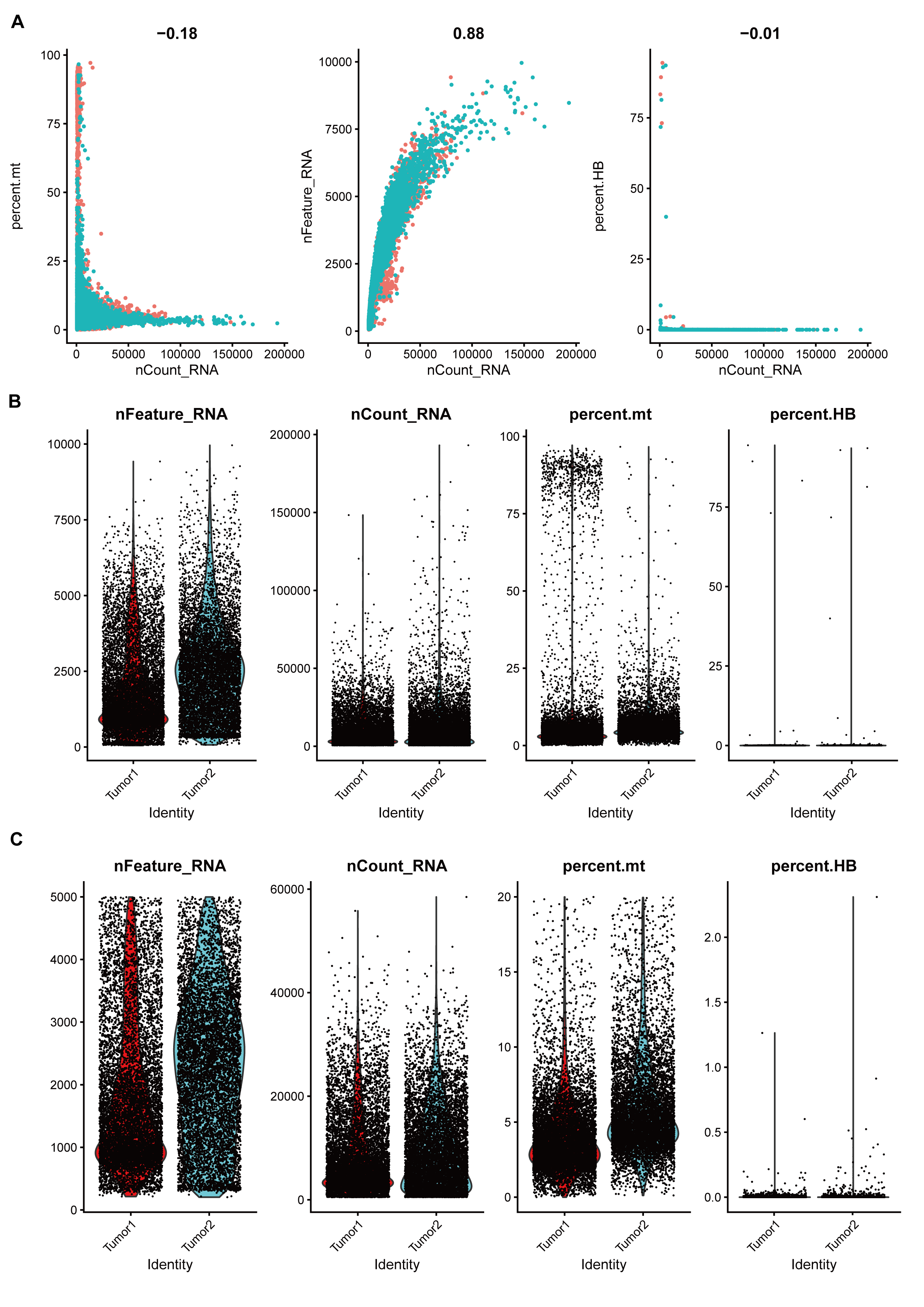
**

**Supplementary Figure 10** | Quality control (QC) of single-cell analysis. **(A)** The relationship between the number of unique molecular identifiers (UMIs) per cell (nCount_RNA) and the percentage of mitochondrial genes per cell (percent.mt), the gene counts per cell (nFeature_RNA), the percentage of hemoglobin genes per cell (percent.HB). **(B, C)** nFeature_RNA, nCount_RNA, percent.mt and percent.HB before and after QC.

**
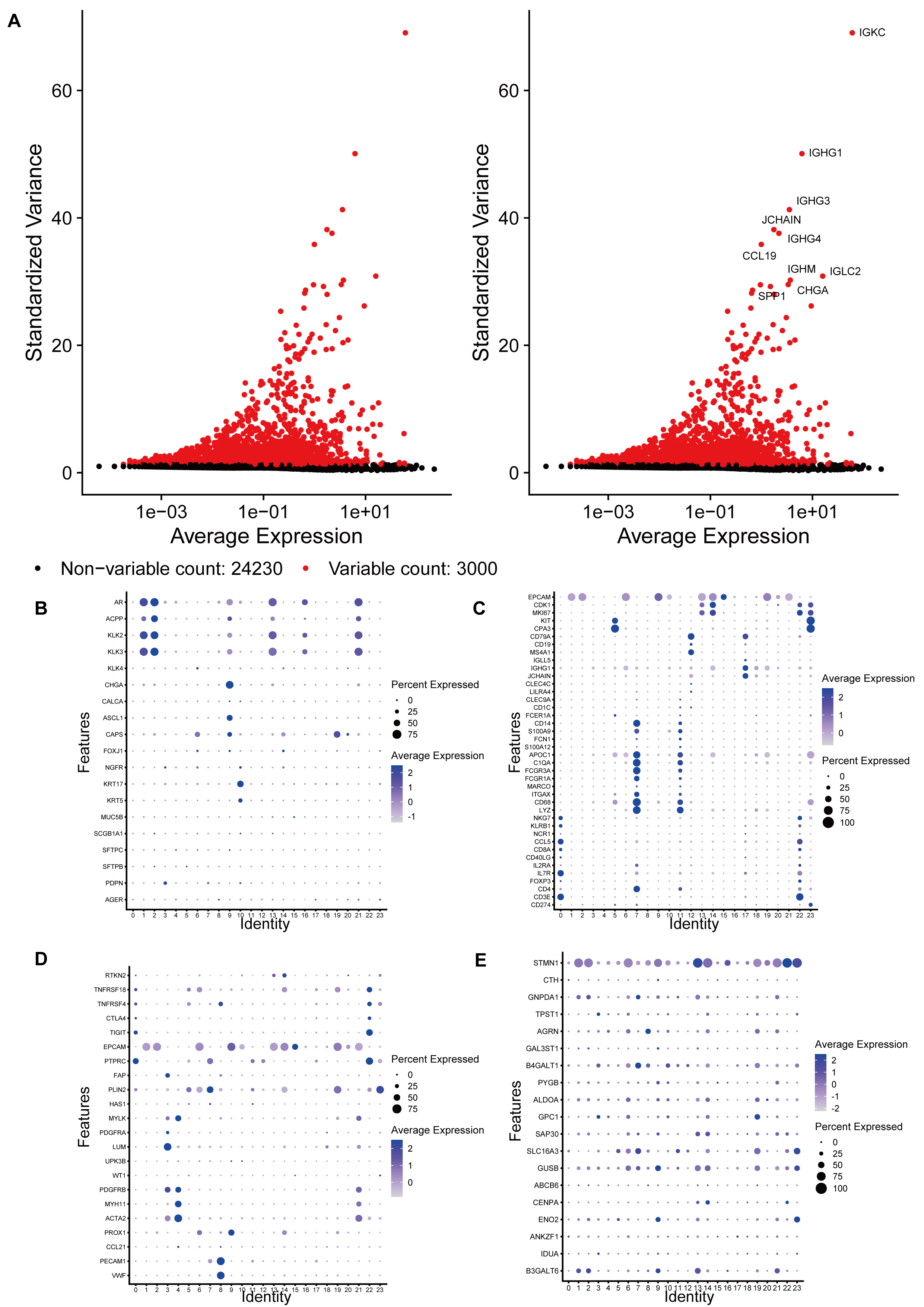
**

**Supplementary Figure 11** | The manual annotation at the single cell level. **(A)** The top 3,000 variable genes were marked in red color, and the 10 most highly variable were labeled. **(B-E)** The different expression of markers for epithelial cells, immune cells, other cells and GRGs in 24 cell clusters.
